# Supplementary material for: The cerebellum promotes sequential foraging strategies and contributes to the directional modulation of hippocampal place cells
Source: iScience. 2023 Feb 15;26(3):106200. doi: 10.1016/j.isci.2023.106200 (PMC10009096; doi:10.1016/j.isci.2023.106200)
Supplement: Document S1. Figures S1–S4 [file mmc1.pdf]

iScience, Volume *26*

Supplemental information

The cerebellum promotes sequential foraging strategies and contributes to the directional modulation of hippocampal place cells

Lu Zhang, Julien Fournier, Mehdi Fallahnezhad, Anne-Lise Paradis, Christelle Rochefort, and Laure Rondi-Reig

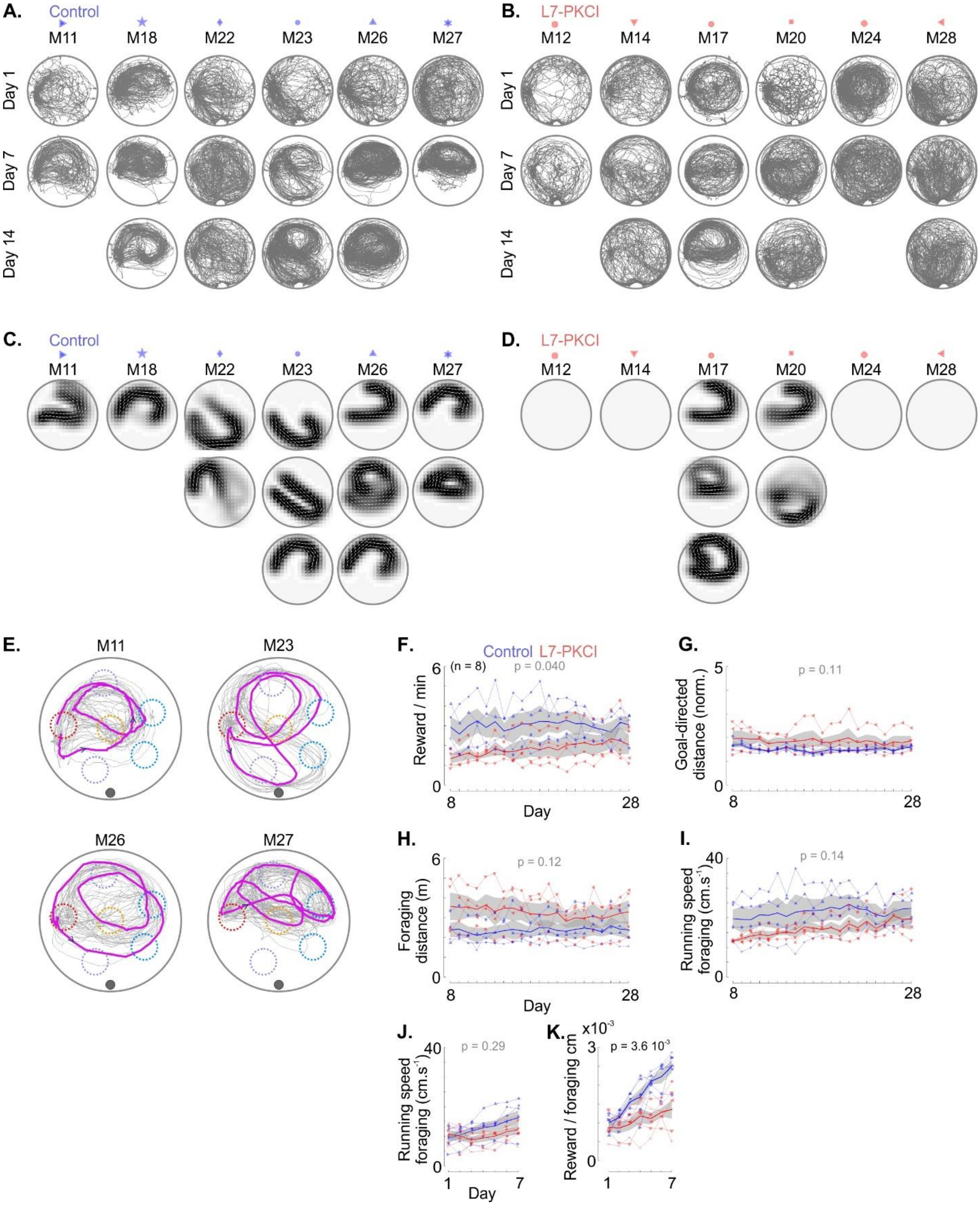

Figure S1: Sequence-based foraging and prolonged training (related to Figure 1).

A. Examples of trials performed by control mice on the 1st (*top*), 7th (*middle*) and 14th (*bottom*) day of training. Symbols indicate mouse identity throughout the figures. **B**. Same as A for L7-PKCI mice. **C.** Templates of spatial sequence identified by hierarchical clustering in all control mice. Columns correspond to different

animals; lines correspond to multiple templates found in the same animal. Symbols indicate mouse identity throughout the figures. **D**. Same as in C for L7-PKCI mice. Blank matrices indicate mice for which no spatial sequence was identified. Arrows indicate the direction of motion. **E.** Example of single-trial trajectories (*purple*) where mice repeated only part of the spatial sequence when they did not obtain the foraging reward. **F.** Number of rewards collected across later days of training (*red*: L7-PKCI, n = 4; *blue*: control, n = 4, mixed GLM, LR test of genotype effect: $\Delta\chi^2(1) = 4.22$, p = 0.04). *Thick curve*: median +/- s.e.m.. **G.** Same as in F for the median distance traveled during the goal-directed phase (mixed GLM, LR test of genotype effect: $\Delta\chi^2(1) = 2.52$, p = 0.11). The goal-directed distance was normalized in each trial by the shortest distance between the position where the animal received the foraging reward and the position where it first entered the goal zone. **H.** Same as in F for the median distance traveled during the foraging phase (mixed GLM, LR test of genotype effect: $\Delta\chi^2(1) = 2.44$, p = 0.12). **I.** Same as in F for the median of running speeds during the foraging phase (mixed GLM, LR test of genotype effect: $\Delta\chi^2(1) = 2.17$, p = 0.14). **J.** Median of running speeds during the foraging phase across the first 7 days of training for L7-PKCI (*red*) and control (*blue*) mice (mixed GLM, LR test of genotype effect: $\Delta\chi^2(1) = 1.25$, p = 0.29). *Thick curve*: median +/- s.e.m.. **K.** Number of reward per minute normalized by the running speed during foraging. This ratio is thus equivalent to a measure of performance per distance unit, which is independent of running speed. L7-PKCI mice still exhibit a lower performance than control mice (mixed GLM, LR test of genotype effect: $\Delta\chi^2(1) = 8.45$, $p = 3.6\ 10^{-3}$), demonstrating that differences in running speed cannot explain the lower performance of L7-PKCI mice.

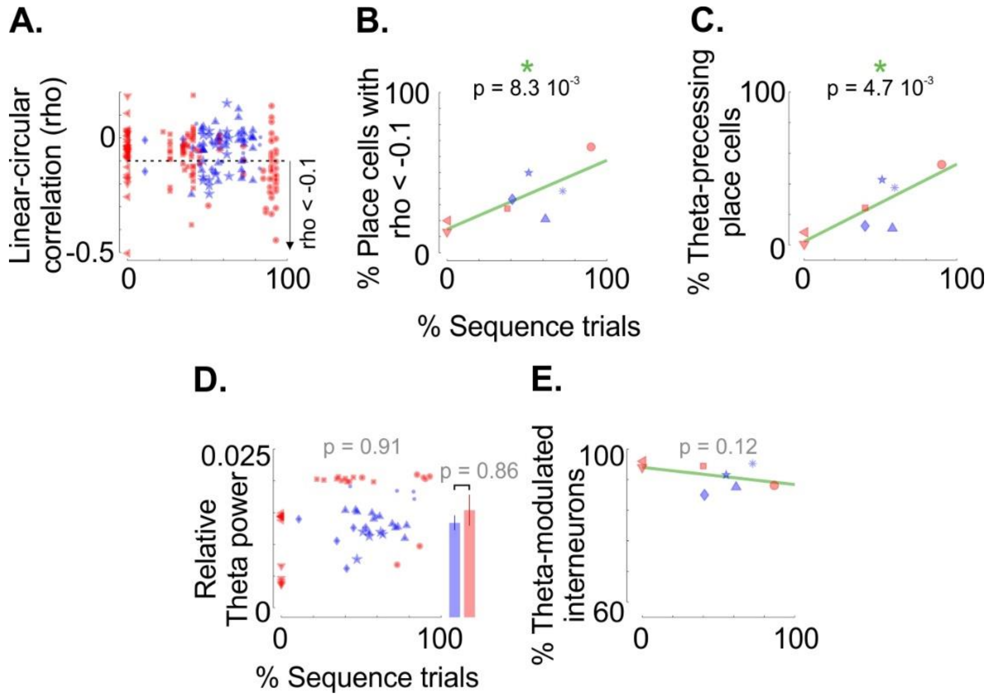

Figure S2. Theta coupling and percentage of sequence trials (related to Figure 2).

A. Linear-circular correlation (rho) as a function of the percentage of sequence trials for all place cells. Dotted line: -0.1 threshold used in B. **B.** The percentage of place cells with a linear-circular correlation coefficient smaller than -0.1 is significantly correlated with the percentage of sequence trials (mixed GLM, LR test for the fraction of sequence trial: $\Delta\chi 2(1) = 6.96$, $p = 8.7\ 10^{-3}$). **C.** Percentage of place cells that exhibited significant theta-phase precession, after selecting only place field traversals that were fast, straight and going through the center of the place field (mixed GLM, LR test for the fraction of sequence trial: $\Delta\chi 2(1) = 7.99$, $p = 4.7\ 10^{-3}$). **D.** Theta power as a function of the percentage of sequence trials for L7-PKCI (*red*) and control (*blue*) mice across recording days (mixed GLM, LR test for the fraction of sequence trial: $\Delta\chi^2(1) = 0.014$ $p = 0.91$). Theta power was measured as the mean power between 6 and 9 Hz, normalized by the sum of the power spectrum across all frequencies up to 40Hz. **E.** Fraction of interneurons modulated by theta phases as a function of the percentage of sequence trials for L7-PKCI (*red*) and control (*blue*) mice (mixed GLM, LR test for the fraction of sequence trial: $\Delta\chi^2(1) = 2.48$, $p = 0.12$).

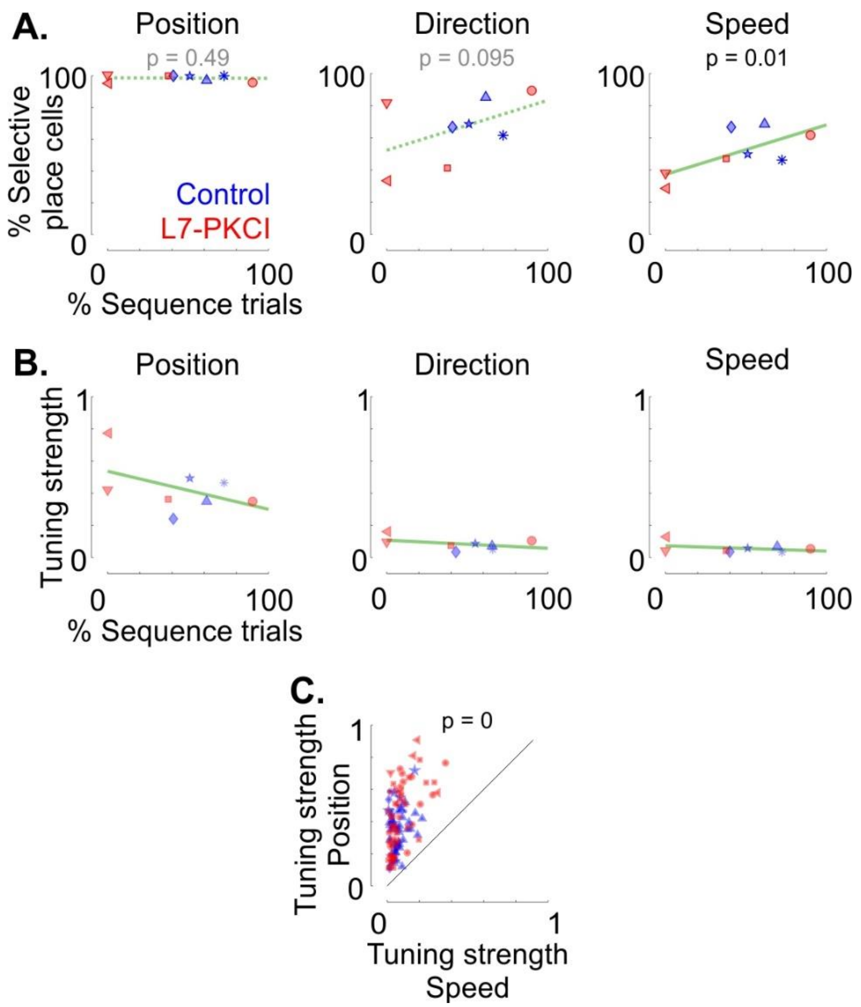

Figure S3. Sequence-based foraging behavior and tuning to positions, direction and speed (related to Figure 3).

A. Fractions of place cells that were identified as selective to spatial positions (*left*), movement direction (*middle*) or speed (*right*) in L7-PKCI (*red*) and control (*blue*) mice, as a function of the percentage of sequence trials. P-values indicate the significance of the effect related to the fraction of sequence trials (mixed GLM, LR test). **B.** Median across place cells of the strength of tuning to positions (*left*), direction (*middle*) and speed (*right*) for L7-PKCI (*red*) and control (*blue*) animals, as a function of the percentage of sequence trials. **C.** Tuning strength to position as a function of the tuning strength to running speed. The p-value indicates the significance of the Position vs Speed effect (mixed GLM, LR test).

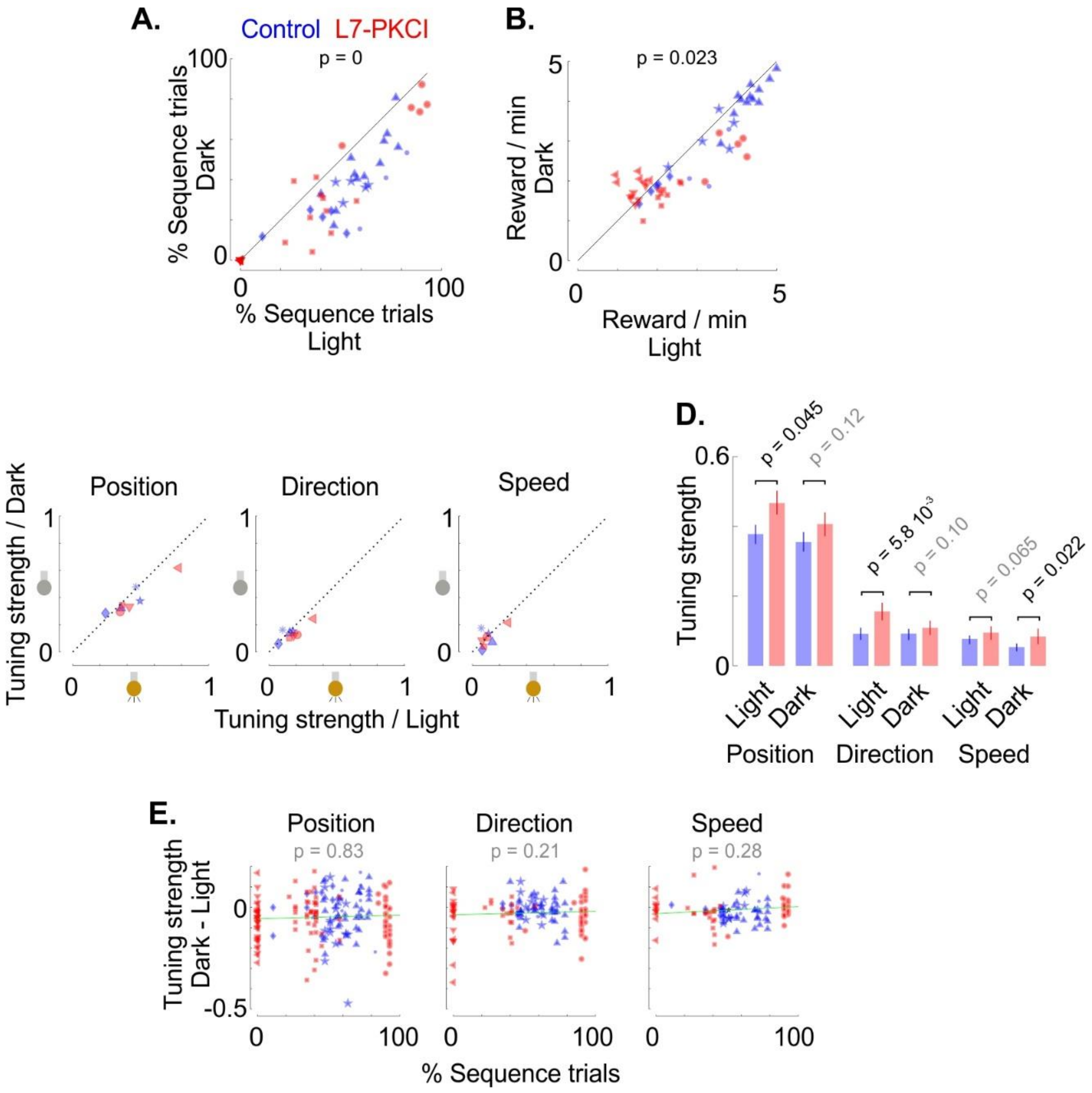

Figure S4. Comparison of performances and place cells tuning between light and dark conditions (related to Figure 4).

A. Comparison of the fraction of sequence trials between dark and light conditions (mixed GLM, LR test for the effect of light/dark condition: $\Delta\chi^2(1) = 210.56$, $p = 0$; LR test for the interaction between light/dark condition and genotype: $\Delta\chi^2(1) = 1.31$, $p = 0.25$). **B.** Comparison of the number of collected rewards between dark and light conditions (mixed GLM, LR test for the effect of light/dark condition: $\Delta\chi^2(1) = 5.10$, $p = 0.023$; LR test for the interaction between light/dark condition and genotype: $\Delta\chi^2(1) = 0.23$, $p = 0.63$). **C.** Comparison of the median across place cells of tuning to position (*left*), movement direction (*middle*) or speed (*right*) between light and dark conditions, for L7-PKCI (*red*) and control (*blue*) mice. **D.** Tuning strength of L7-PKCI and control place cells for position, direction and speed during light and dark conditions. P-values indicate the significance for the genotype effect (mixed GLM, LR test). **E.** Difference between the tuning strength measured in the dark and in the light (Dark - Light) as a function of the percentage of sequence trials for Position, Direction and Speed tuning. P-values indicate the significance for the interaction between the effect of the light/dark condition and the percentage of sequence trials (mixed GLM, LR test).
